# Induction of Sphk1 activity in obese adipose tissue macrophages promotes survival

**DOI:** 10.1371/journal.pone.0182075

**Published:** 2017-07-28

**Authors:** Tanit L. Gabriel, Mina Mirzaian, Berend Hooibrink, Roelof Ottenhoff, Cindy van Roomen, Johannes M. F. G. Aerts, Marco van Eijk

**Affiliations:** 1 Department of Medical Biochemistry, Academic Medical Center, Amsterdam, The Netherlands; 2 Department of Biochemistry, Leiden Institute of Chemistry, Leiden University, Leiden, The Netherlands; 3 Department of Cell Biology, Academic Medical Center, University of Amsterdam, Amsterdam, the Netherlands; University of Cambridge, UNITED KINGDOM

## Abstract

During obesity, adipose tissue macrophages (ATM) are increased in concert with local inflammation and insulin resistance. Since the levels of sphingolipid (SLs) in adipose tissue (AT) are altered during obesity we investigated the potential impact of SLs on ATMs. For this, we first analyzed expression of SL metabolizing genes in ATMs isolated from obese mice. A marked induction of sphingosine kinase 1 (*Sphk1*) expression was observed in obese ATM when compared to lean ATM. This induction was observed in both MGL-ve (M1) and MGL1+ve (M2) macrophages from obese WAT. Next, RAW264.7 cells were exposed to excessive palmitate, resulting in a similar induction of *Sphk1*. This *Sphk1* induction was also observed when cells were treated with chloroquine, a lysosomotropic amine impacting lysosome function. Simultaneous incubation of RAW cells with palmitate and the Sphk1 inhibitor SK1-I promoted cell death, suggesting a protective role of Sphk1 during lipotoxic conditions. Interestingly, a reduction of endoplasmic reticulum (ER) stress related genes was detected in obese ATM and was found to be associated with elevated *Sphk1* expression. Altogether, our data suggest that lipid overload in ATM induces *Sphk1*, which promotes cell viability.

## Introduction

White adipose tissue (WAT) acts as the major body storage for fat from which free fatty acids (FFAs) can be mobilized [1]. FFAs are used as energy supply during starvation and for heat production, but also act as building blocks for various essential lipids such as steroids, eicosanoids, (phospho)glycerolipids and SLs [2]. Over-nutrition and sedentary life promote an increase of WAT mass and adipocyte size, favouring obesity and imbalanced homeostasis of metabolism. During obesity, adipocyte necrosis takes place due to excessive ER stress and hypoxia [3]. Subsequently, excessively FFAs are released into the circulation together with chemokines and pro-inflammatory cytokines, triggering further local recruitment of inflammatory cells in the adipose tissue. This cascade promotes a pro-inflammatory environment, which drives insulin resistance (IR) and type-2 diabetes [4]. The adaptive and innate immune system both contribute to the chronic low grade inflammatory status of obese WAT. Changes in virtually all immune cells have been associated to obese adipose tissue derailment, including T cells, neutrophils, Natural Killer (NK) cells, dendritic cells, NK T cells, invariant NK T cells, eosinophils, mast cells and B cells [5–14]. The most dramatically in numbers increased immune cell in obese WAT is the ATM [15]. Moreover, during obesity the ATM undergoes a change in phenotype. Obese ATMs are polarized towards a more classically activated (M1) state and are associated with lipid accumulation, whereas in lean WAT alternatively activated (M2) ATMs prevail [13,14,16]. Interestingly, treatment with rosiglitazone in mice induced a redistribution of lipids towards adipocytes and reduced the presence of M1 polarized ATM [16]. Recently a potential role for lysosomes in the phenotypic change of obese ATMs has been recognized. Induction of lysosome biogenesis in these cells has been observed, a process also excited by lysosomal stress [17]. In line with this, we recently found glycoprotein nonmetastatic melanoma protein B (Gpnmb) to be induced in obese ATM as a consequence of lipid-induced lysosomal stress [18]. Of interest, lysosomal stress also occurs in inherited disorders in intralysosomal sphingolipid catabolism, either caused by a deficiency in a lysosomal glycosphingolipid hydrolase such as glucocerebrosidase in Gaucher disease or by a generalized lysosome dysfunction as in Niemann-Pick type C disease. Both in Gaucher disease and Niemann-Pick type C disease Gpnmb has also been found to be markedly induced [19–21]. These findings suggest that impaired lysosomal sphingolipid metabolism induces lysosome biogenesis and might be considered to also occur in obese ATMs.

There exist numerous SL species with various functions, ranging from structural membrane components to bioactive lipid mediators [22]. SL synthesis universally begins with the condensation of serine to palmitoyl-CoA at the cytosolic leaflet of the ER generating sphinganine (Spha) [23]. Next, Spha acylation is carried out by ceramide synthases (CerS), generating dihydroceramides (DHCer) that are converted into ceramides (Cer) by the action of a desaturase (DES). Excessive Cer is known to induce programmed cell death. Normally, it serves as building block for more complex SLs such as sphingomyelin (SM), ceramide 1-phosphate (Cer-1-P) and the carbohydrate containing glycosphingolipids (GSLs) [24]. The catabolism of complex GSLs resides largely in lysosomes and depends on stepwise hydrolysis of the terminal sugar moieties [23]. The formed Cer is next deacylated by acid ceramidase to generate the bioactive molecule sphingosine (Spho), which is either transported to the ER and re-enters the SL cycle being transformed to ceramide (salvage pathway) or phosphorylated by sphingosine kinases (Sphk) 1 or 2 to generate sphingosine-1-phosphate (S1P) [25–27]. S1P can be exported or dephosphorylated again by a phosphatase to Spho. However, most S1P is transformed by S1P lyase (SPL1) to phosphoethanolamine and trans-2-hexadecenal [28]. Oxidation of the latter by the long change aldehyde dehydrogenase (ALDH3a2) renders palmitoleic acid that is further catabolized by mitochondrial fatty acid beta-oxidation.

To study the role of SL in observed ATM changes during obesity, we first analyzed gene expression profiles of different SL synthesizing -and degrading enzymes in sorted ATM from both genetic and diet-induced obese (DIO) mouse models. *Sphk1* gene expression was found to be strikingly induced in obese ATM from both models. This induction was observed in both MGL-ve (M1) and MGL1+ve (M2) macrophages from obese WAT. Concomitantly, Sphk1 activity was found to be elevated. Exposing cultured RAW 264.7 macrophage-like cells to high amounts of palmitate also induced *Sphk1* gene expression and activity. Finally, we provide evidence that Sphk1 plays a critical role in macrophage survival during lipotoxic conditions.

## Materials and methods

### Cell culture experiments and reagents

RAW264.7 obtained from the American Type Culture Collection were cultured in DMEM/10% FCS supplemented with penicillin-streptomycin. Palmitic acid (Sigma) was coupled to BSA Fraction V fatty acid—free (Roche) as described previously [18] and used at 250 and 500 μmol/L. Interleukin (IL)-4 (R&D Systems), IL-10 (PeproTech), and interferon-γ (IFN-γ; PeproTech) were used at 50 ng/mL; lipopolysaccharide (LPS) from *Salmonella minnesota R 595* (Alexa) was used at 100 ng/mL; chloroquine (CQ) was used at 40 μmol/L (Sigma); tunicamycin at 10 μg/mL (Sigma); the Sphk1 inhibitor, SK1-I 12.5 μmol/L (Enzo Life Sciences).

### Animals

12-week-old male C57BL/6J control mice (WT) and leptin-deficient obese (ob/ob) mice (C57BL/6J background) were purchased from Harlan laboratories (Horst, Netherlands). The animals were fed ad libitum commercial chow diet (AM-II) (Arie Blok BV, Woerden, the Netherlands). 6-week-old male C57Bl/6J mice were fed ad libitum a high fat diet (HFD; D12492, Research Diets Inc.; consisting of 20 kcal % protein, 20% carbohydrates, and 60% fat) or low-fat diet (LFD; D12450B, Research Diets Inc.; consisting of 20 kcal % protein, 70% carbohydrates, and 10% fat) for 16 weeks. Principles of laboratory animal care were followed. All animal protocols were approved by the Institutional Animal Welfare committee of the Academic Medical Center in the Netherlands (#DBC101943). The mice (n = 6 per group unless stated otherwise) were housed in a temperature-controlled room on a 12-h light-dark cycle.

### Flow cytometry and CD11b^+^ selection

ATM were FACS sorted from the stromal vascular fraction (SVF) of epididymal fat pads as has been described previously [18]. Briefly, SVF was incubated with rat anti-mouse CD16/CD32 mouse Fc—blocking reagent (BD Biosciences) for 10 min. Subsequently, SVFs were double labelled with rat anti-mouse macrophage galactose—specific lectin 1 (MGL1)/CD301 Alexa 647 (Bio-Connect) and rat anti-mouse F4/80 fluorescein isothiocyanate (eBioscience) for 25 min. Using a FACSAria instrument (BD Biosciences) MGL1^+^/F4/80^+^ [M2] (from WT, LFD, ob/ob and HFD SVFs) and MGL1^lo^/F4/80^+^ [M1] (from ob/ob and HFD SVFs) cells were sorted. MGL1^lo^/F4/80^+^ [M1] were present at insufficient levels to perform analysis in WT and LFD mice.

To obtain CD11b^+^ cells, SVFs were incubated with anti-CD11b conjugated magnetic microbeads and enriched according to the manufacturer’s protocol (Miltenyi Biotec). CD11b^+^ selected cells were labelled using anti-mouse F4/80 fluorescein isothiocyanate and analyzed using a FACSCalibur instrument (BD Biosciences) to assess macrophage purity.

### Sphingolipid quantification and Sphk1 activity assay

Ceramide (Cer) and glycosphingolipid (GSL) levels were determined by HPLC as described previously [29]. Shortly, after adding C17-sphinganine (Avanti) as internal standard, lipids were extracted according to Bligh/Dyer et al [30]. Subsequently, the chloroform phase was thoroughly dried, and deacylation of lipids was performed in 0.5 mL of 0.1 M NaOH in methanol in a microwave (CEM microwave Solids/Moisture System SAM-255) for 60 min. Deacylated lipids were derivatized on-line for 30 min with Phtaldialdehyde (Sigma/Aldrich) and separated with a Ultra High Pressure Liquid Chromatography system with fluorescence detection (UHPLC Thermo Scientific) and a Alltima C_18_ 3 μm, 150 mm x 4.6 mm reverse-phase column (Grace Davison Discovery Science Inc., USA). All samples were measured in triplicate, unless otherwise stated, and in every run a reference sample was included.

Sphingosine-1-phosphate (S1P) detection and quantification was performed by liquid chromatography coupled to tandem mass spectrometry (LC-MS-MS). C17 base S1P (Avanti) was used as internal standard. After a total extraction and protein precipitation with chloroform-methanol 1:2 (v/v), the solvents were evaporated at 30°C by Eppendorf concentrator 5301. The residue was extracted with butanol/water 1:1 (v/v), the upper phase was dried and dissolved in 100μL methanol.

Spho, Spha and sphingosin-dien were measured by LC-MS-MS after methanol/chloroform extraction in acidified conditions methanol:chloroformand:100mM ammonium formate buffer pH 3.15; 1:1:0.9 (v/v/v) using C17-sphinganine as internal standard [31].

Sphk1 activity was measured as described previously [32]. Briefly, after CD11b^+^ enrichment, or culture of RAW 264.7, cells were washed three times with PBS, and disrupted by one freeze/thaw cycle and 20 passages thought a 25 gauge needle in Sphk1 assay buffer (20 mM Tris-HCl (pH 7.4), 1 mM EDTA, 20% glycerol, 1 mM 2-mercaptoethanol, 1 mM Na-orthovanadate, 40 mM β-glycerophosphate, 15 mM NaF, 0.5 mM 4-deoxypyridoxine, and protease inhibitor cocktail (Roche Applied Science, EDTA free)). In low salt and 0.25% Triton X-100, Sphk2 activity is negligible [26]. After protein determination the assay was performed in a glass tube using 60 μgr of sample, which was incubated with 50 μM Sphingosine (Avanti), 0.25% Triton X-100, 10 mM MgCl_2_ and 1.1 mM ATP (Roche) for 60 minutes at 37°C. The reaction was stopped by adding chloroform:methanol, a different extraction method was used to remove the Spho excess. After protein precipitations, lipids were extracted from the supernatant the supernatant according to Blight and Dyer method [30]. Phase separation extraction with chloroform:methanol:water; 1:1:0.9 (v/v/v) was required. The upper phase was dried and further extracted by butanol:water; 1:1 (v/v), the butanol phase was taken to the dryness and the residue was dissolved in 100μL methanol. Finally, S1P was determined by LC-MS-MS as previously described.

### RNA isolation and Q-PCR analysis

Total RNA was extracted from FACS-sorted ATMs, or CD11b^+^ enriched cells using TRIzol (Invitrogen) reagent and the NucleoSpin II extraction kit following manufacturer’s instructions (Macherey Nagel). NanoDrop spectrophotometer (NanoDrop Technologies) was used to determine RNA concentrations. Equal amounts of RNA were used to synthesize cDNA according to the manufacturer’s method (Invitrogen). iCycler MyiQ system (Bio-Rad) was used for real-time Q-PCR to analyse gene expression. Expression levels in mouse were normalized to those of acidic ribosomal phosphoprotein 36B4, also referred to as P0. Analysis of the relative changes in gene expression was performed using the 2^-ΔΔ*C*T^ method. List of primers used for the Q-PCR are in the S1 Table. Gene expression analysis for the various markers could not be followed up by confirmation at the level of protein due to the limited yield after cell enrichment procedures.

### Western blot analysis

After stimulations of cells with palmitate, or BSA vehicle alone, cells were washed three times with PBS and total cell lysates were prepared in RIPA lysis buffer (150 mmol/L NaCl, 50 mmol/L Tris-HCl pH 7.4, 2 mmol/L EDTA, 0.5% deoxycholaat, 1 mmol/L Na_3_VO_4_, 20 mmol/L NaF, 1 mM PMSF and 0.5% Triton X-100) supplemented with protease inhibitor cocktail (Roche). Lysates were incubated for 30 min on ice and subsequently centrifuged at 4°C for 10 min at 14.000x g. Samples were separated on a 7.5% SDS gel and transferred to nitrocellulose membranes. Primary antibodies used: rabbit anti-PARP (Cell signalling; #9542) and mouse anti- alpha tubulin (Cedarlane Laboratories Limited).

### Cell viability assay

Cell viability was determined using the WST-1 assay (Roche) following the manufacturer’s instructions. Absorbance at 450 nm was determined using an ELISA plate reader (Synergy; Bio Tek). WST-1 is a metabolic activity indicator and therefore should be interpreted with caution.

### Heat-map and cluster analysis

Hierarchical cluster analysis was performed using relative gene expression levels, which were transformed to Z-score values (mean centered and scaled by standard deviation), using the HierarchicalClustering module from GenePattern software [33]. Genes were clustered using the pairwise complete linkage method, in which distance are the calculated Pearson’s correlation coefficient. Fold change (log2) values of lipids in CD11b^+^ enriched were represented in a heat map using Genepattern software.

### Statistical analysis

All results are expressed as mean ± S.D. The statistical significance of differences between groups was analyzed using unpaired Student *t*-test or Whitney-Mann as indicated.

## Results

### *Sphk1* expression is increased in obese ATM from HFD and ob/ob mice

**Fig 1:**
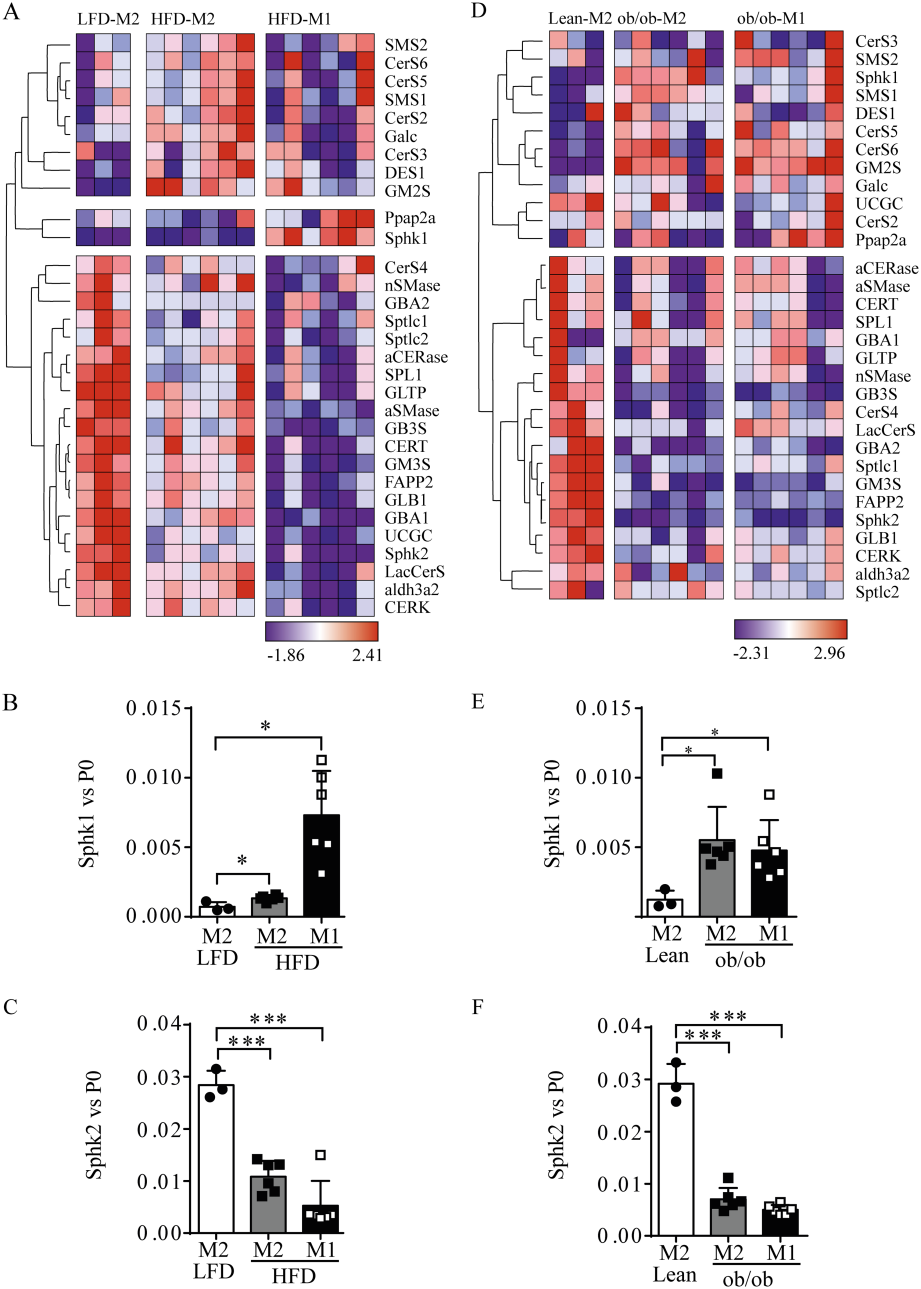
Hierarchical cluster analysis (vertical) of expression of genes involved in (glyco)sphingolipid metabolism and transport in FACS sorted ATM populations. A. LFD ATMs (F4/80-Mgl1^+^[M2]) (n = 9 mice, 3 mice were pooled together for the analysis) and HFD ATMs (both F4/80-Mgl1^+^ [M2] and F4/80-Mgl1^-^ [M1]) (n = 6). The red color represents high-normalized expression levels and the blue color represents low expression levels. B. Relative *Sphk1* and C. *Sphk2* gene expression levels from LFD vs HFD ATM populations normalized by acidic ribosomal phosphoprotein 36B4 (P0). D. Hierarchical cluster analysis in WT, lean ATM (F4/80-Mgl1^+^[M2]) (n = 9 mice, 3 mice were pooled together for the analysis) and obese ob/ob ATM (both F4/80-Mgl1^+^ [M2] and F4/80-Mgl1^-^ [M1]) (n = 6 mice). E. Relative *Sphk1* and F. *Sphk2* gene expression levels lean vs. ob/ob relative to P0. Whitney-Mann test was used to evaluate significance in gene expression; * p < 0.05; *** p < 0.001.

To get insight in the role of GSL metabolism in obese ATM, a selected set of genes involved in GSL synthesis, breakdown (S1 Fig) and transport was analyzed by quantitative PCR (QPCR) in sorted ATM populations isolated as described previously [14,18]. LFD fed mouse ATM (F4/80^+^ MGL1^+^: 'M2') and HFD fed mouse ATM (F4/80^+^ MGL1^-^: 'M1') and (F4/80^+^ MGL1^+^: 'M2') were studied. Hierarchical cluster analysis of the gene expression z-scores revealed that three clustered families can be observed in the mice challenged with HFD and LFD (Fig 1A). The first group comprises 9 genes, which are elevated in HFD F4/80^+^ MGL1^+^ ATM and reduced in F4/80^+^ MGL1^-^ ATM from HFD and F4/80^+^ MGL1^+^ ATM from LFD fed mice. The second group consists of genes displaying elevated expression in LFD F4/80^+^ MGL1^+^ ATM and reduced expression in both ATM populations isolated from the HFD mice. The third group is composed of two genes, *Sphk1* and phosphatidic acid phosphatase type 2A (*Ppap2a*), which are highly elevated in HFD fed mice F4/80^+^ MGL1^-^ ATM. Considering that Sphk1 was the gene most prominently induced in sorted obese macrophages compared to lean/LFD models and given the pivotal role of Sphk1 activity for instance in pancreatic β-cell survival during lipotoxic conditions, we focussed on Sphk1 regulation and role in obese ATM [34,35].

The relative expression levels of *Sphk1* in the ATM populations are depicted in more detail in a graph (Fig 1B). Remarkably, the isoenzyme *Sphk2* behaves in the opposite direction in the sorted ATM of HFD animals (Fig 1C).

A comparable gene expression analysis was performed focussing on obese ATM populations isolated from the genetic, leptin-deficient ob/ob mouse model. The hierarchical cluster analysis is depicted in Fig 1D. In sorted ATM from lean mice a comparable profile was observed, when compared to the LFD group. Importantly, in sorted ob/ob F4/80^+^ MGL1^-^ATM *Sphk1* was highly expressed as well (Fig 1D). The relative gene expression of *Sphk1* in sorted ob/ob ATM is depicted in the graph in Fig 1E. As for the HFD ATM populations *Sphk2* levels are also in the opposite direction, so higher in the lean group and reduced in ob/ob ATM populations (Fig 1F).

### Sphk1 activity is increased in ob/ob WAT enriched CD11b^+^ cells

**Fig 2:**
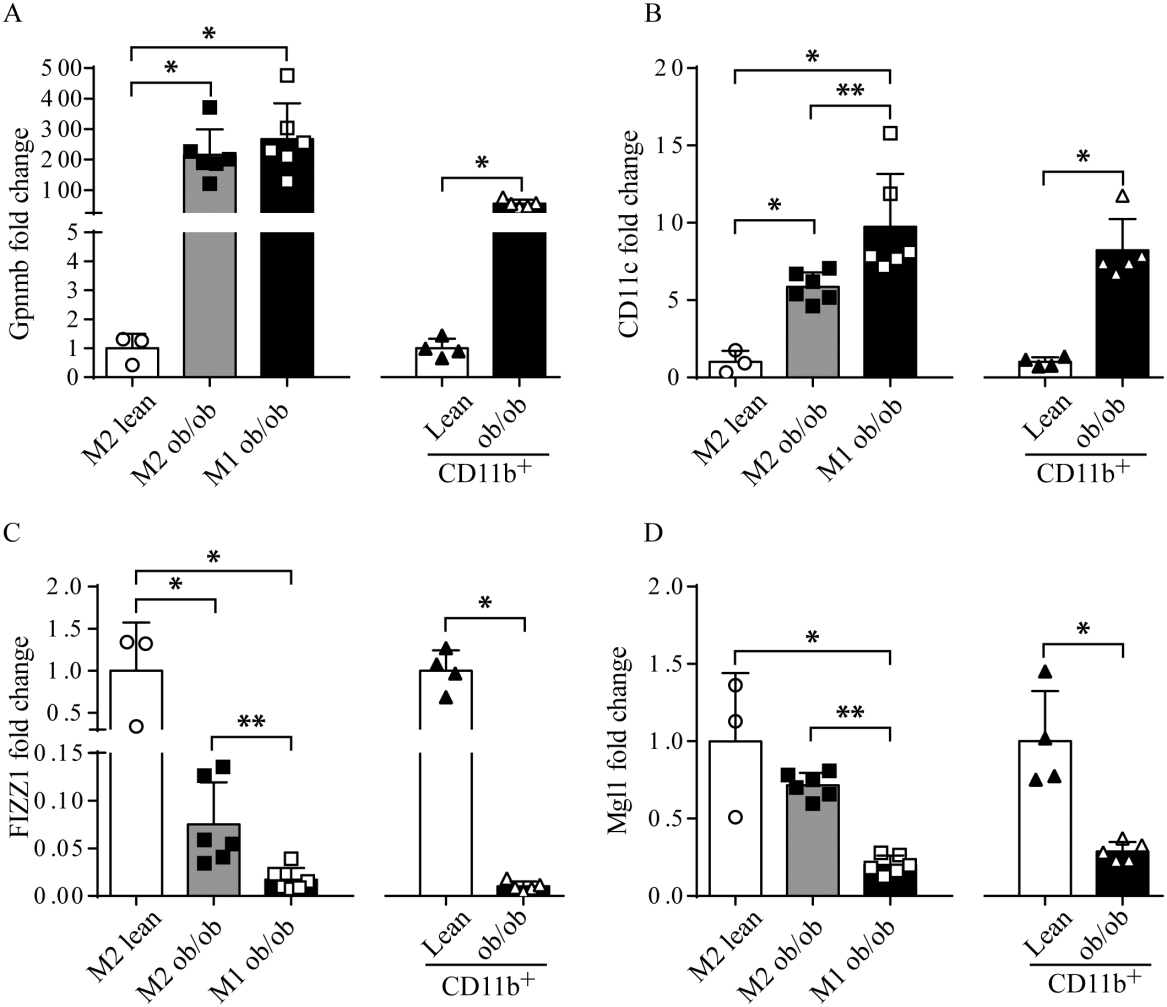
Characterization of FACS sorted and CD11b^+^ isolated ATM phenotype. A. mRNA levels of the macrophage markers *Gpnmb*, B. *CD11c*, C. *FIZZ1* and D. *Mgl1* were analyzed by QPCR in CD11b^+^ enriched cells and compared to FACS sorted ATM from lean and ob/ob WAT. Whitney-Mann test was used to evaluate significance in gene expression; * p < 0.5; ** p < 0.05.

Next we addressed if the increase in *Sphk1* gene expression also translated into an increase in Sphk1 activity. To increase ATM yield we enriched for macrophages by positively selecting CD11b^+^ cells from the SVF of lean and obese ob/ob WAT using anti-CD11b antibodies coated with magnetic beads (MACS) [36]. First we analyzed the phenotype of the CD11b^+^ enriched cells. FACS analysis revealed that the CD11b^+^ populations where >90% positive for F4/80 indicating that the majority of the cells are macrophages. Moreover, QPCR gene expression analysis revealed that the CD11b^+^ enriched cells and sorted ATM displayed similar induction of *Gpnmb* and *CD11c* expression (Fig 2A and 2B). *Fizz1* and *Mgl1*, both M2 macrophage markers, are down regulated to the same extent in obese CD11b^+^ cells when compared to sorted ob/ob ATM (Fig 2C and 2D). The enrichment for CD11b^+^ cells provided sufficient yield to determine Sphk1 enzyme activity and analysis of lipid content.

**Fig 3:**
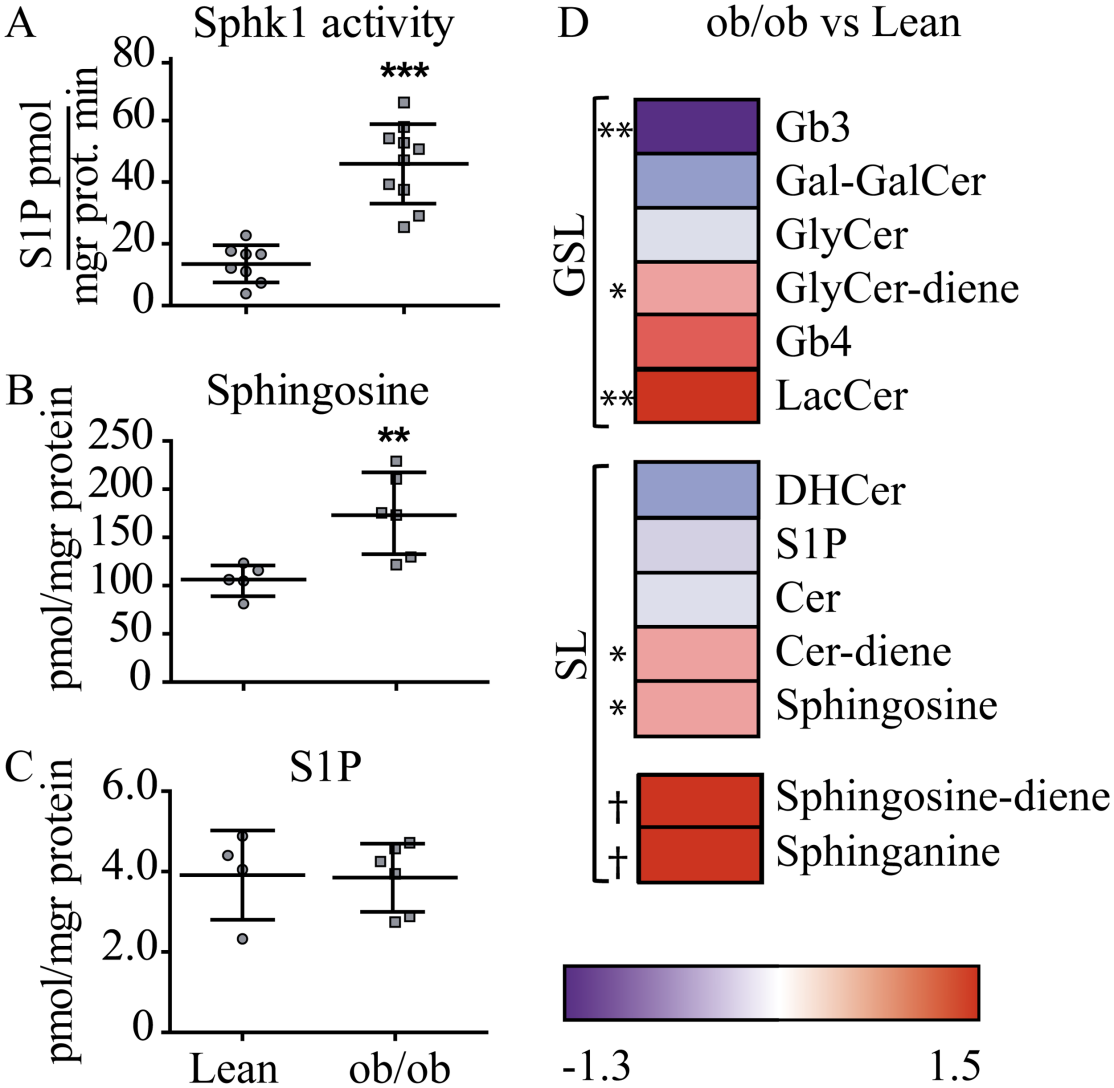
(G)SL and Sphk1 activity analysis in CD11b^+^ ATM isolated from ob/ob and lean mice SVF. A. Sphk1 activity. B. Sphingosine and C. S1P levels. Data depicted in the plots are analyzed using the Whitney-Mann test. D. Fold change (log2) values of GSL analyzed in WAT ob/ob vs lean CD11b^+^ macrophages are represented in a heat map (n = 4 and n = 6 in respective lean and ob/ob). The red color represents high-normalized levels and the blue color represents low levels of the lipids. Significance is evaluated by Whitney-Mann test and indicated; * p < 0.05; ** p < 0.01*** p < 0.001; † detected only in ob/ob CD11b^+^ cells and not included in the heat map analysis.

In line with the increase in *Sphk1* gene expression in FACS sorted ob/ob ATM, we found a marked increase of Sphk1 activity in CD11b^+^ enriched cells isolated from ob/ob SVF (Fig 3A). Additionally, lipid analysis revealed that the Sphk1 substrate and S1P precursor, Spho, was significantly elevated in ob/ob CD11b^+^ cells (Fig 3B). S1P levels from ob/ob macrophages were in the same range as the lean cells (Fig 3C).

Interestingly, further lipid analysis showed that ob/ob ATM present a different lipid signature compared to WT. The sphingoid bases, Spho, sphingosine-diene and Spha are increased together with the GSL lactosylceramide (LacCer) and glycosylceramide-diene (GlyCer-diene) (Fig 3D). The GSL globotriaosylceramide (Gb3) was significantly reduced in ob/ob CD11b^+^ ATM, whereas several analyzed lipid species were not significantly changed when compared to the lean cells (Fig 3D).

### Sphk1 gene expression and activity are induced in macrophages by palmitate and CQ

**Fig 4:**
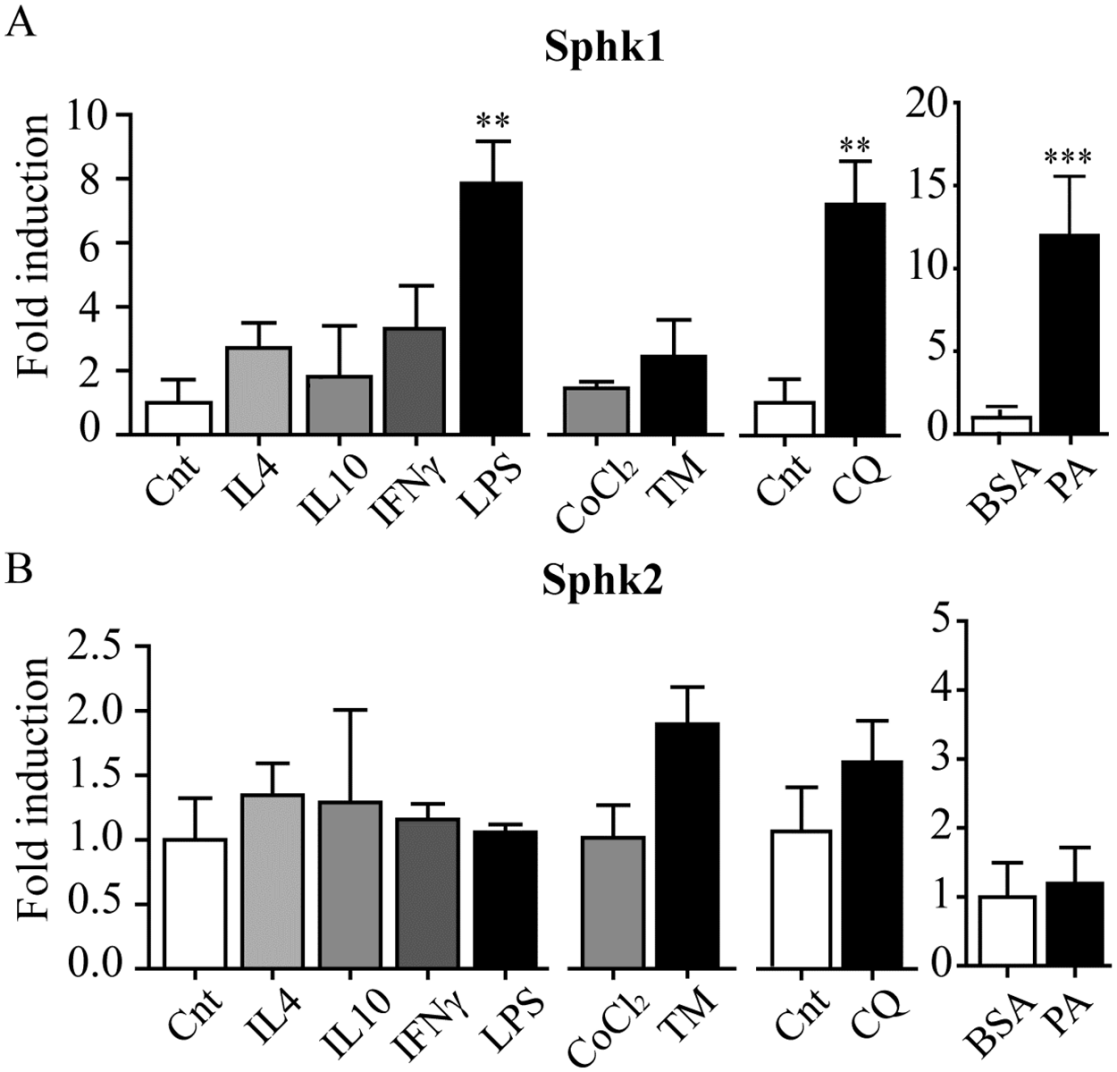
*Sphk1* expression is induced by LPS, palmitate and chloroquine (CQ). A. *Sphk1* and B. *Sphk2* gene expression levels after 4h stimulation of RAW 264.7 with inflammatory mediators, hypoxia inducing CoCl2, ER stress inducing tunicamycin (TM), the lysosomal stressor CQ and 24 hours 500 μM BSA coupled palmitate (PA). Data are depicted as mean +/- S.D. (n = 3 per group). * p < 0.05; ** p < 0.01; *** p < 0.001.

It is known that various factors during obesity are altered. Not only the inflammatory status of the AT switches from a T helper cell type 2(Th2)-like (T cell-originated IL-10 and eosinophil IL-4) to a Th1-like environment (T cell-derived IFN-γ), but also metabolic changes occur such as hypoxia, ER stress, lipotoxicity induced by FFA and more recently described lysosomal stress [18,37,38]. To get more insight in the regulation of *Sphk1* expression and its activity in obesity, we systematically tested above conditions using the mouse macrophage-like RAW264.7 cells. We first studied inflammation by investigating the anti-inflammatory cytokines IL-4 and IL-10 and the pro-inflammatory factors IFN-γ and LPS. (Fig 4A). In line with earlier work we found that LPS induced *Sphk1* gene expression [39]. Efficacy of the inflammatory stimuli was verified by analysing *TNF-α* and *arginase-1* (S2 Fig). Neither hypoxia, induced by using CoCl2, nor ER stress, induced using tunicamycin (TM) resulted in elevation of *Sphk1* gene expression (Fig 4A). Efficacy of the stimuli was again confirmed. CoCl2 potently induced *GLUT1* expression and *CHOP* was strongly induced with TM. We recently found that mimicry of lipotoxicity using palmitate induced *Gpnmb*, a marker induced upon lysosomal stress [18]. Potent induction of *Sphk1* gene expression was found by both palmitate and the lysosomal stressor chloroquine (CQ) (Fig 4A). Efficacy of the stimuli was verified by Gpnmb induction (S2 Fig). In parallel *Sphk2* gene expression was also determined and was found not to be induced in the conditions tested (Fig 4B).

**Fig 5:**
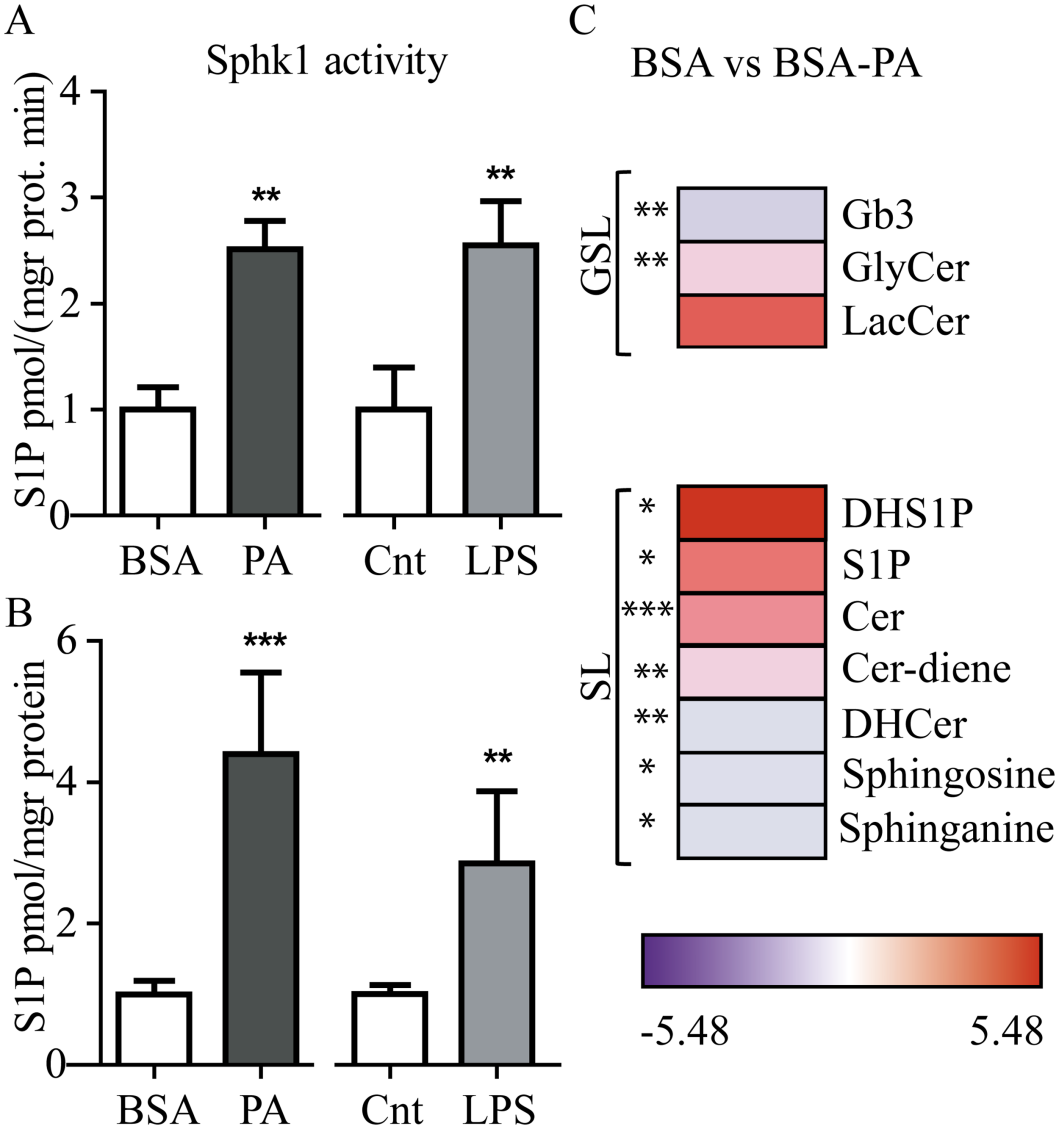
Sphk1 activity and S1P analysis in palmitate and LPS treated RAW264.7 cells. A. Sphk1 activity and B. S1P analysis normalized to respective controls. C. Fold change (log2) values of (G)SL analyzed in Raw264.7 challenged with BSA vs 500 μM BSA coupled to Palmitate (BSA-PA). The red color represents high-normalized levels and the blue color represents low levels of the lipids. (n = 3). Significance is evaluated by a student's t-test and indicated as * p < 0.05; ** p < 0.01 and ** p < 0.001.

In agreement with the gene expression data, Sphk1 activity was increased in RAW264.7 challenged with palmitate and LPS (Fig 5A). Next we analyzed S1P levels under the same conditions and observed that palmitate and LPS induced S1P levels (Fig 5B). Interestingly, also other (G)SL species analyzed showed an increase upon palmitate treatment (Fig 5C).

### Sphk1 protects macrophages from lipid induced apoptosis

**Fig 6:**
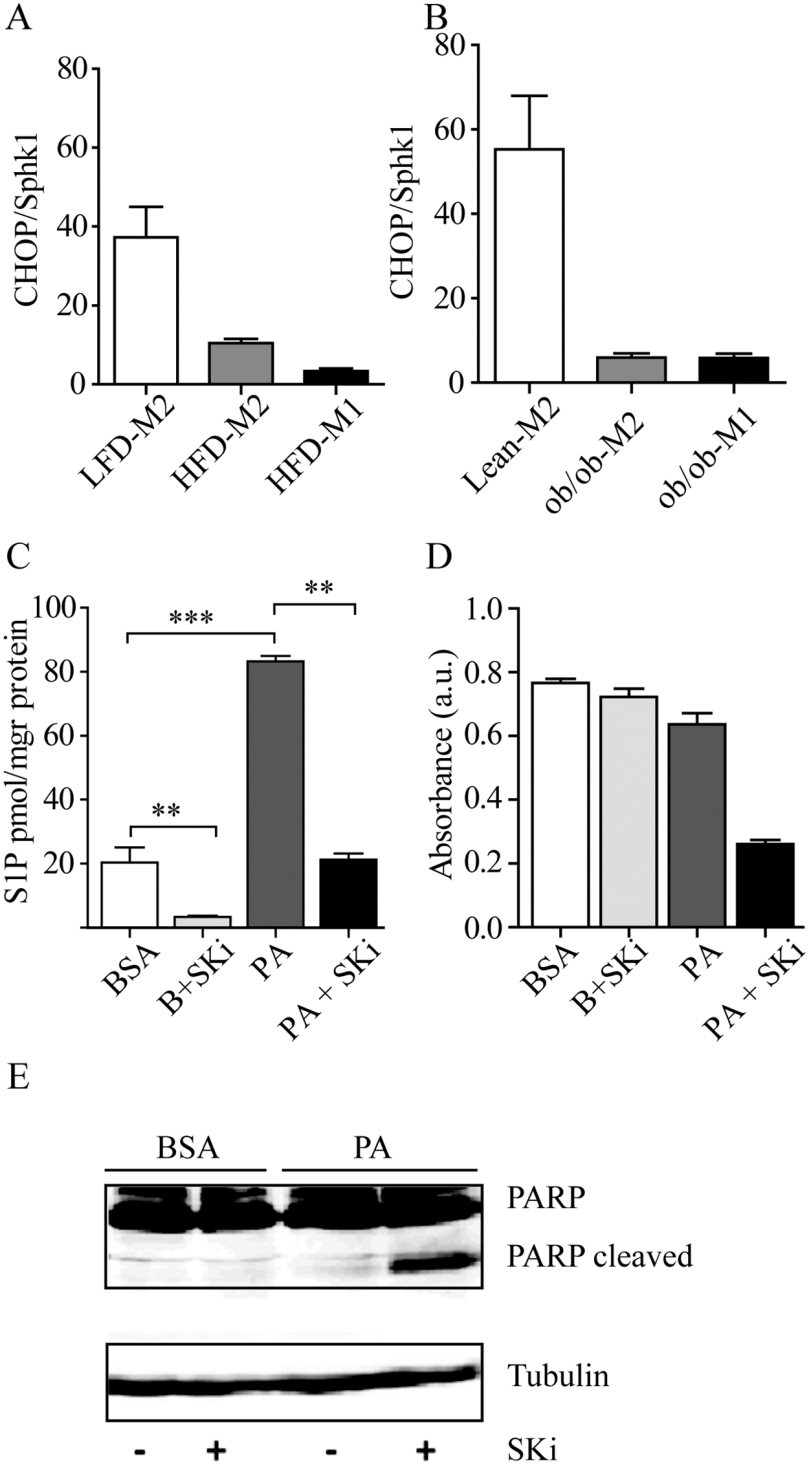
Sphk1 activity contributes to cell survival upon challenge of RAW264.7 cells with palmitate and chloroquine. The *CHOP/Sphk1* ratio is reduced in FACS sorted ATM A. HFD ATMs (both F4/80-Mgl1^+^ [M2] and F4/80-Mgl1^-^ [M1]) and B. ob/ob ATMs (both F4/80-Mgl1^+^ [M2] and F4/80-Mgl1^-^ [M1]). C. The *CHOP/Sphk1* ratio is also reduced in CD11b^+^ enriched cells from SVF D. S1P analysis showed that SK1-I inhibits Sphk1 activity. Analysis of the role of Sphk1 in promoting cell viability using E. WST-1 assay E. PARP cleavage analyzed by western blot in RAW264.7 cells challenged with 250 μM BSA coupled palmitate in the presence or absence of the Sphk1 inhibitor SK1-I for 24h (WST-1), or 8 h (PARP cleavage). Data in the graphs are depicted as mean +/- S.D. (n = 3) ** p < 0.01; *** p < 0.001.

Lipotoxicity induces ER stress amongst others characterized by the activation of the C/EBP homologous protein (CHOP)-dependent unfolded protein response pathway [40]. Furthermore, previous work showed that dysregulation of sphingolipid metabolism induces ER stress via CHOP upregulation [41]. We observed an increased Spho versus S1P ratio in obese ATM (Fig 3D). Furthermore, gene expression analysis in FACS sorted ATM revealed that the ratio of *CHOP/Sphk1* is decreased in both the HFD fed and ob/ob ATM populations (Fig 6A and 6B), suggesting that obese macrophages may be protected from ER stress and associated apoptosis. To test if Sphk1, thus reducing Ceramide and/or Spho, contributes to survival, RAW264.7 were challenged with BSA coupled palmitate in the presence of the Sphk1 inhibitor SK1-I. Analysis of S1P levels showed that levels are indeed decreased in cells incubated with SK1-I (Fig 6C). By using different assays it is demonstrated that Sphk1 activity is crucial for survival upon challenge with palmitate. In the presence of SK1-I, thus no Sphk1 activity, cell survival is severely reduced after overnight incubation using WST-1 as a measure for cell survival (Fig 6D). Furthermore, it was found that PARP was already processed after 8h when SK1-I was used to block Sphk1 activity (Fig 6E). In the presence of CQ, SK1-I also perturbed cell viability (S3 Fig). Taken together the data this suggests that Sphk1 activity is required for protection of ATM against FFA-induced cell death.

## Discussion

It is widely accepted that the risk of developing insulin resistance during obesity is associated with altered SL metabolism [42,43]. Changes in the innate and adaptive immune system during obesity also are known to cause inflammation of adipose tissue [44]. Macrophages in the adipose tissue spectacularly increase in number and show a more M1 prone phenotype [15,45]. The present study aimed to identify a possible link between abnormal SL metabolism and ATM during obesity. For this, we first comparatively analyzed in sorted ATM populations from normal, ob/ob and HFD fed mouse the expression of genes involved in SL metabolism.

Obese ATM showed down regulation of most SL metabolism genes as compared to lean M2 ATM. However expression Sphk1 mRNA was most prominently induced in obese ATM in comparison to lean ATM. Moreover, we confirm increased Sphk1 activity in obese CD11b^+^ enriched ATM. In line with this finding, elevated Sphk1 expression in total AT in obese subjects and mice has previously been reported [42,46,47]. Interestingly, a lack of differential expression of *Sphk1* in ob/ob M1 and M2 macrophages was observed, whereas M1 ATM from HFD presented an increased expression compared to M2. Even though the genetic background between all the groups were the same, they present intrinsic differences. ob/ob are leptin-deficient, younger and have a different diet compared to the HFD group. Other studies reported that aging alone already has an impact on sphingolipid composition in organs such as brain [48]. Furthermore, HFD model animals increase weight during a defined period when the diet is offered, whereas the ob/ob mice have been dealing with their condition since pups, allowing for adaptive mechanisms. Alternatively the diet itself is strongly associated with obesity and can have a considerable impact on metabolic regulation, possibly affecting Sphk1 regulation in M1 and M2 macrophages. Interestingly, the Sphk1 isoform, *Sphk2*, was found down regulated in obese ATM from both models. Currently it is unknown by what mechanism this is triggered, but several studies also describe that reduced *Sphk2* is accompanied by induced *Sphk1*. Gao et al. showed in tumour cells that Sphk1 ablation did not display any effects on Sphk2 expression and S1P levels [49]. Other studies using different models, namely, tumour cells, acute colitis and knock out mice in which *Sphk2* expression was depleted, showed an increase of *Sphk1* expression, activity and S1P levels [46,47,50,51]. The Spiegel group demonstrated that the molecular mechanism of *Sphk1* induction by *Sphk2* downregulation is histone deacetylase HDAC1/2 activity and c-Jun dependent [51]. In addition, it has been shown in RAW264.7 that palmitate reduced HDAC activity and induced c-Jun [52,53]. Altogether these reports suggest that the *Sphk1* increase observed in obese ATM might in part be due to a reduction of the *Sphk2* expression levels. The observed induction of *Sphk1* in obese ATM was recapitulated in macrophage-like RAW264.7 cells exposed to a high amount of palmitate, hence mimicry of a lipotoxic environment. This was not accompanied by a decrease in *Sphk2* expression. The reason for this is not understood, but possibly the ratio of *Sphk1* vs *Sphk2* is relevant in this context. It has been previously reported that *Sphk1* expression is also induced by palmitate in C2C12 myocytes, primary hepatocytes and HepG2 challenged with FFA [54,55]. In parallel, we confirmed that palmitate feeding to RAW264.7 cells also promotes Sphk1 activity. Of note, we observed in obese ATM an elevation of Spho, the substrate of sphingosine kinases. No concomitant increase in S1P was detected, suggesting that either S1P is rapidly secreted, or degraded through S1P lyase activity followed by beta-oxidation, or both. Earlier, Samad et al. demonstrated that plasma S1P levels were increased in ob/ob mice, yet S1P levels were hardly detectable in AT [42]. Further studies reported that S1P levels are increased in human plasma from diabetic obese individuals [42,56]. The majority of the circulating S1P is bound to the apolipoprotein M (ApoM) in the high-density lipoprotein (HDL) [57]. Furthermore, secretion of S1P by the obese AT and down regulation of S1P degrading enzymes has been reported, again suggesting that great part of the S1P generated in the tissue is secreted [56]. In our gene expression analysis from the sorted ATM, we show that crucial genes involved in S1P degradation; *SPL1* and *aldh3a2* are down regulated during obese conditions (Fig 1A and 1D). Taken all together, our results suggest that *in vivo* most of the S1P generated by the cells is likely to be secreted and released into the extracellular compartment, rather than being degraded. Interestingly, it has been reported in obese mice that treatment with rosiglitazone induced changes in ceramide levels in ATM and triggered redistribution of lipids towards adipocytes, improving insulin sensitivity in obese animals and a reduced presence of M1 polarized ATM, underscoring the association between lipid alterations and M1 ATM polarization [16]. Lastly, we explored the biological role of Sphk1 activity in macrophages undergoing a high FFA pressure, hence mimicking local adipocyte-derived FFA elevation in obesity. Unlike Cer and Spho, a relative increase in S1P levels promotes cell survival and inhibits apoptosis [58]. The control of cell fate depends on the balance between S1P on one hand, and on ceramide and Spho on the other hand, which is known as the sphingolipid biostat/rheostat [59]. It is reported that increased Sphk1 activity and consequently increase S1P/Cer-Spho, plays a critical role in pancreatic β-cell survival under lipotoxic conditions [34,35]. In addition, it recently has been demonstrated in dendritic cells that S1P generated by Sphk2 activity is uncoupled from cell survival, since Sphk2 activity failed to rescue *Sphk1* deficient cells that were exposed to LPS [60]. Moreover, it has been shown that *Sphk1* overexpression in muscle counteracts Cer levels and ameliorates insulin resistance in HFD mice corroborating the role of Sphk1 in cell survival [61]. Furthermore, Boslem et al. demonstrated that glucosylceramide synthase overexpression protects pancreatic β-cells from palmitate driven CHOP induction and apoptosis by increasing the ratio of glucosylceramide/Cer, ergo reducing Cer and Spho levels [62]. Interestingly, the above discussed rosiglitazone effects in ob/ob mice coincided with reduced ceramide species containing palmitate as a fatty acid group (Cer(d18:1/16:0)) [16]. In line with those studies, our data on increased gene expression and Sphk1 activity *in vivo*, suggests a shift towards S1P production counteracting the apoptosis supposedly promoted by Spho and Cer. Additionally, our gene analysis showed a reduction of *CHOP* expression in the obese ATM, indicating that those cells are protected from the lipid spill over by the adipocytes. Importantly, when RAW264.7 cells were treated simultaneously with the Sphk1 inhibitor (SK1-I) and palmitate, cell death induction was not prevented, suggesting an essential protective role of Sphk1 during lipotoxic conditions in macrophages. Consistently, Hill et al. showed recently that obese ATM present an enhanced survival phenotype through a mechanism involving NF-kB activation [63]. Interestingly, it has also been demonstrated that Sphk1 seems not to be required for inflammatory responses in macrophages, reinforcing the idea of Sphk1 playing an essential pro-survival role in obese ATM [64].

In summary, our data suggest that lipid-mediated lysosomal stress induction promotes Sphk1 activity in obese ATM, favouring macrophage survival, thus allowing the generation of a local lipid sink in AT and thereby contributing to the prevention of acute systematic lipotoxicity.

## Supporting information

S1 Table List of Q-PCR primers used for gene expression analysis. (XLS)

S1 Fig A schematic representation of the sphingolipid metabolic pathway studied. Gene name abbreviations, encoding for sphingolipid metabolic enzymes are in grey boxes and color code square boxes indicate expression levels of the enzymes measured in different macrophage population from ob/ob and lean mice. Quantified sphingolipid metabolites from ob/ob vs lean CD11b^+^ cells are in rounded cornered colored boxes containing sphingolipid names, the black boxes are sphingolipids not analyzed. Arrows indicate the direction of the reaction. Sphinganine (Spha), DhCer (Dihydroceramides), Cer (Ceramides), Cer1P (Ceramide-1-phosphate), GlcCer (Glucosylceramide), LacCer (Lactoylceramide), GM3 (Monosialodihexosylganglioside 3), GM2 (Monosialodihexosylganglioside 2), Cer1P (Ceramide-1-phosphate), SM (sphingomyeline), Spho (Sphingosine), S1P (Sphingosine-1-phosphate), DhS1P (Dihydrosphingosine-1-phosphate), EP (phosphoethanolamine).
(TIF)

S2 Fig TNF, Arg-1, glut-1, CHOP and Gpnmb expression levels in RAW264.7 upon different conditions. TNF was strongly induced by LPS and INFγ (A). IL4 induced Arg-1 (B) and Glut-1 was induced by CoCl2(C). ER stress marker CHOP was strongly induced by tunicamycin (TM) and Gpnmb was induced by palmitate (E).
(TIF)

S3 Fig Sphk1 activity contributes to cell survival upon challenge of RAW264.7 cells with chloroquine CQ. WST-1 assay A. PARP cleavage analyzed by western blot in RAW264.7 cells challenged with 40 μmol/L CQ in the presence or absence of the Sphk1 inhibitor SK1-I for 8h.
(TIF)
