# Supplementary figures and images for: Induction of Sphk1 activity in obese adipose tissue macrophages promotes survival
Source: PLoS One. 2017 Jul 28;12(7):e0182075. doi: 10.1371/journal.pone.0182075 (PMC5533446; doi:10.1371/journal.pone.0182075)

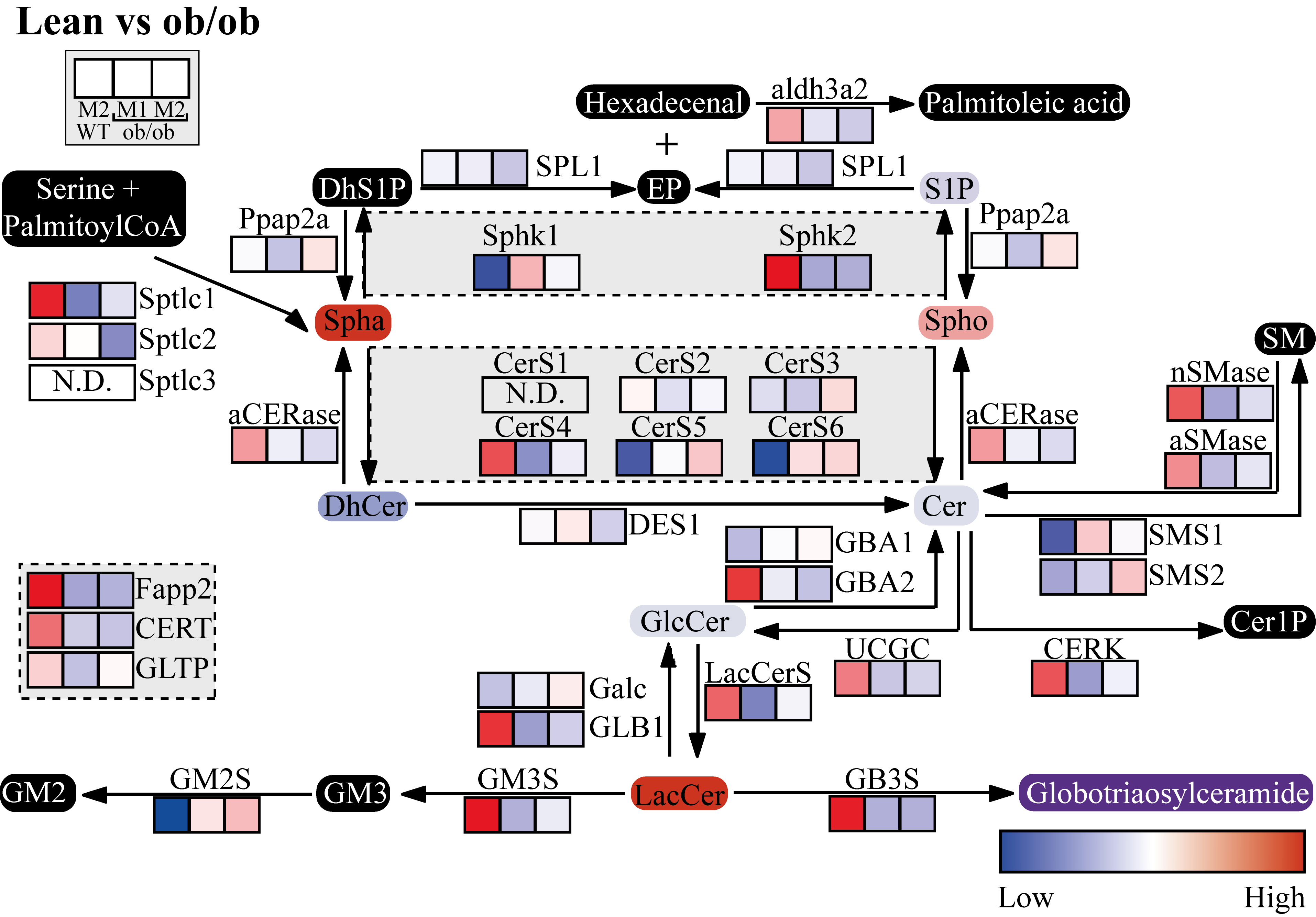

Supplement: S1 Fig — Gene name abbreviations, encoding for sphingolipid metabolic enzymes are in grey boxes and color code square boxes indicate expression levels of the enzymes measured in different macrophage population from ob/ob and lean mice. Quantified sphingolipid metabolites from ob/ob vs lean CD11b+ cells are in rounded cornered colored boxes containing sphingolipid names, the black boxes are sphingolipids not analyzed. Arrows indicate the direction of the reaction. Sphinganine (Spha), DhCer (Dihydroceramides), Cer (Ceramides), Cer1P (Ceramide-1-phosphate), GlcCer (Glucosylceramide), LacCer (Lactoylceramide), GM3 (Monosialodihexosylganglioside 3), GM2 (Monosialodihexosylganglioside 2), Cer1P (Ceramide-1-phosphate), SM (sphingomyeline), Spho (Sphingosine), S1P (Sphingosine-1-phosphate), DhS1P (Dihydrosphingosine-1-phosphate), EP (phosphoethanolamine). (TIF) [file pone.0182075.s002.tif]

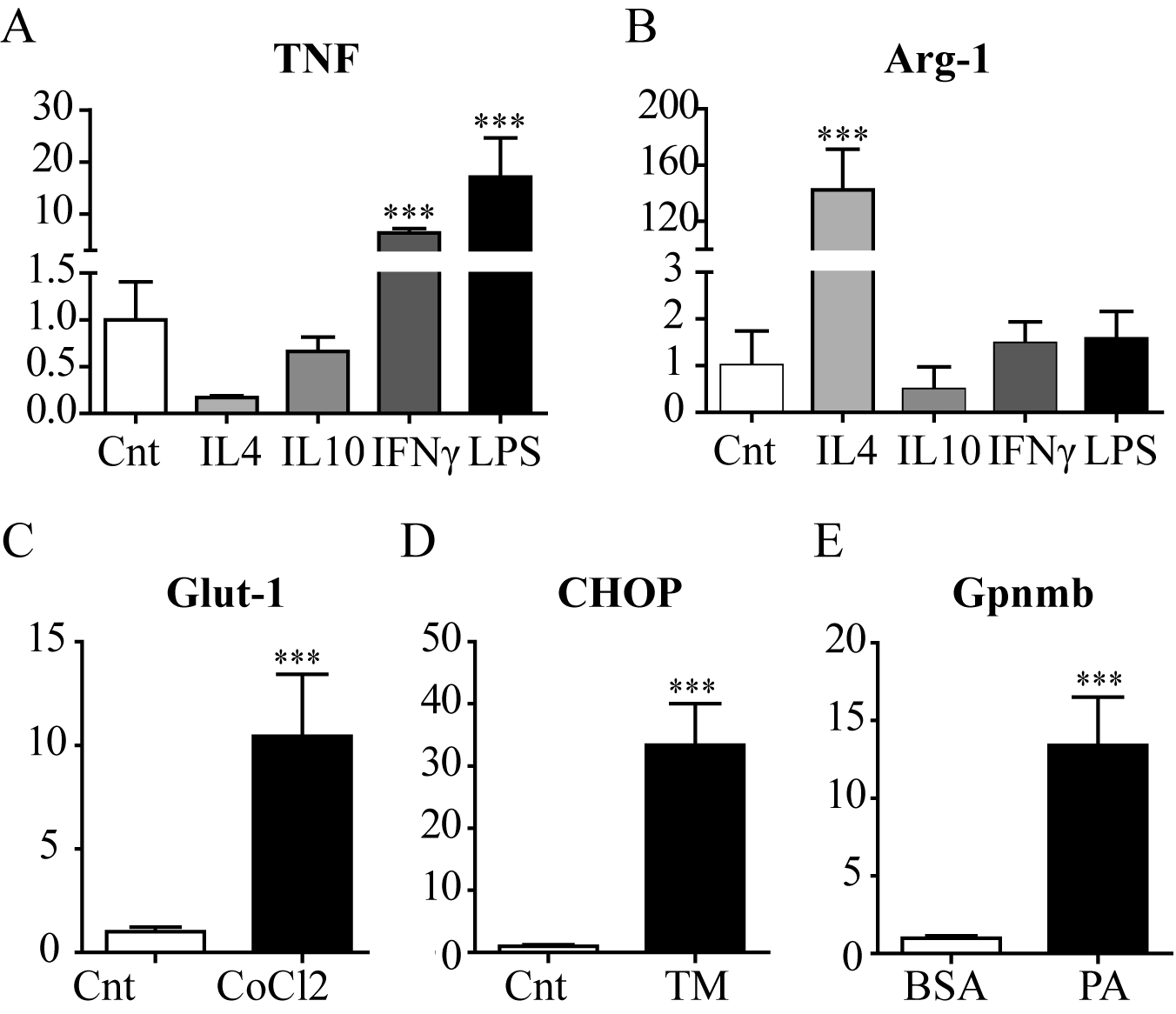

Supplement: S2 Fig — TNF was strongly induced by LPS and INFγ (A). IL4 induced Arg-1 (B) and Glut-1 was induced by CoCl2(C). ER stress marker CHOP was strongly induced by tunicamycin (TM) and Gpnmb was induced by palmitate (E). (TIF) [file pone.0182075.s003.tif]

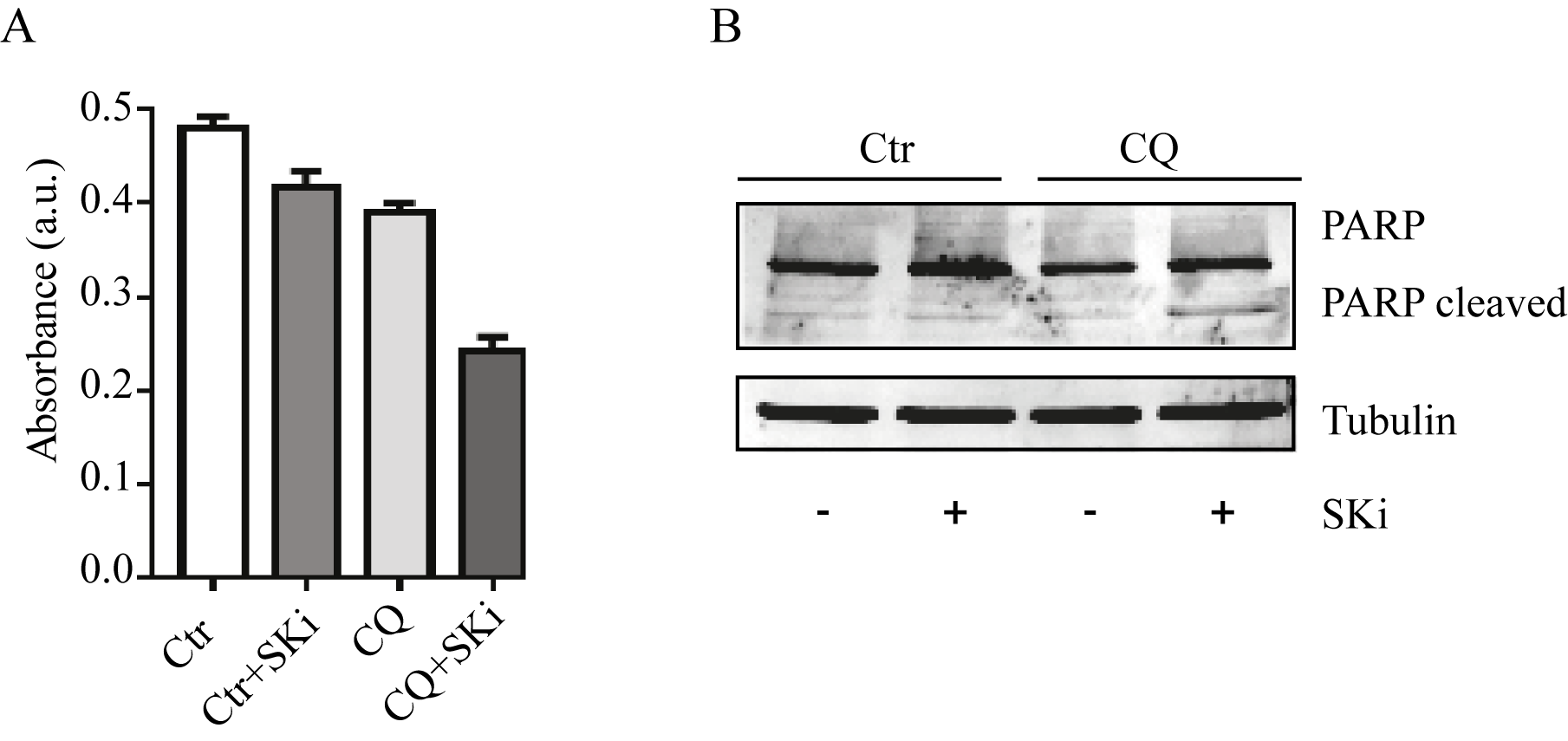

Supplement: S3 Fig — WST-1 assay A. PARP cleavage analyzed by western blot in RAW264.7 cells challenged with 40 μmol/L CQ in the presence or absence of the Sphk1 inhibitor SK1-I for 8h. (TIF) [file pone.0182075.s004.tif]
